# Trends in Incidence of Two Major Subtypes of Liver and Bile Duct Cancer: Hepatocellular Carcinoma and Cholangiocarcinoma in Songkhla, Southern Thailand, 1989-2030

**DOI:** 10.1155/2018/8267059

**Published:** 2018-12-23

**Authors:** Seesai Yeesoonsang, Edward McNeil, Shama Virani, Surichai Bilheem, Chakrarat Pittayawonganon, Chuleeporn Jiraphongsa, Hutcha Sriplung

**Affiliations:** ^1^Epidemiology Unit, Faculty of Medicine, Prince of Songkla University, Thailand; ^2^Office of the Permanent Secretary, Ministry of Public Health, Thailand; ^3^Thailand MOPH-US CDC Collaboration (TUC), Ministry of Public Health, Thailand

## Abstract

**Background:**

The incidence of liver and bile duct cancer continues to rise, especially in Thailand. We aimed to project the trends in incidence of this rare but lethal cancer in southern Thailand in order to determine its future disease burden.

**Methods:**

Gender-specific trends in age-standardized incidence rates per 100,000 person-years for hepatocellular carcinoma (HCC) and cholangiocarcinoma (CCA) cases in Songkhla province of southern Thailand diagnosed between 1989 and 2013 were estimated and projected up to year 2030 using three different modeling techniques: a joinpoint model, an age-period-cohort model, and a modified age-period-cohort model.

**Results:**

Of 2,676 liver and bile duct (LBD) cancer cases identified, 73% were males, 51% were aged between 50 and 69 years, and HCC (44.4%) was slightly more common than CCA (38.1%). The models all predicted an increase in the incidence rate of CCA up to 2025 for both sexes whereas the incidence of HCC is expected to decrease among males and stabilize among females. The incidence rates of HCC and CCA among males in 2030 could reach 6.7 and 9.4 per 100,000 person-years, respectively, whereas the expected rates of HCC and CCA among females are expected to be around 1.5 and 3.9 per 100,000 person-years, respectively.

**Conclusions:**

The incidence of cholangiocarcinoma is expected to increase in Songkhla and will contribute a larger proportion of LBD cancers in the future. Future public health efforts and research studies should focus on this increasing trend.

## 1. Introduction

Liver and bile duct (LBD) cancers were the second leading cause of cancer deaths worldwide in 2012 [1]. In Thailand, liver cancers have the highest incidence in men and the third highest incidence in women [2]. Distinguished by different histological characteristics and cells of origin, LBD cancer is classified into two major histological types: hepatocellular carcinoma (HCC) and cholangiocarcinoma (CCA).

The HCC incidence varies widely across the world due to the distribution of the common risk factors: hepatitis B and C viral infection, aflatoxin exposure, and alcohol consumption [3]. In Thailand, hepatitis C virus has a low prevalence [4]. However, due to the success in reducing HCC in Taiwan and Singapore [5, 6] by introducing hepatitis B virus (HBV) vaccinations in the mid-1980s to the early 1990s, Thailand began a national HBV newborn vaccination program in 1992 [7]. However, some years before the launch of the program, a portion of uninfected children and adults voluntarily vaccinated themselves. Aflatoxin B_1_ exposure is still prevalent in the Thai population due to consumption of brown rice and peanuts [8, 9]. A review showed that aflatoxin is probably among the causes of HCC in the Thai population [10].

CCA is much less common than HCC worldwide; however, there is a high incidence of CCA in Thailand [11]. Recently, an increase in the incidence of CCA has been observed in many countries such as England [12], other European countries [13], and the United States [14]. Established risk factors include parasitic infections, primary sclerosing cholangitis, biliary-duct cysts, hepatolithiasis, and toxins [15]. The incidence of CCA in Thailand has historically been linked to infection of a species of the liver fluke,* Opisthorchis viverrini* (OV), which is especially prevalent in the northeast [11]. Alcohol consumption was reported as one of the risk factors of CCA in a study in northeastern Thailand [16].

Songkhla, a province in the south of Thailand, is situated on the eastern side of the Malay Peninsula adjoining Malaysia to the south and, thus, approximately 25% of the population is Muslim. The proportion of LBD cancer in Songkhla was very low among the primary liver cancer in the past [17], the incidence of LBD cancer has been increasing in the past decade as published in our previous study [18]. Till the early 21^th^ century, the OV infestation in southern Thai population has still a very low prevalence [11]; thus, the continuous increase in incidence of CCA in Songkhla during the recent decades is unlikely to be related to OV infestation. People in the southern region of Thailand do not have the habit of eating raw fish which is the route of OV infection. The latest report on the distribution of* Bithynia siamensis goniomphalos* snail, the first intermediate host of OV fluke stated that there is still no such a snail in southern Thailand [19].

From 2000 to 2012, while there was a decline in Thailand's national average age-standardized incidence rates of liver cancer in both genders [17], the incidence rates of liver cancer in southern Thailand increased from 8.5 to 13.6 in males and 3.3 to 3.8 in females. Liver cancer is a growing problem in the southern region; however, trends by histology have not yet been determined. The scarcity of the reports on trends by histology in many countries is largely due to the high number of cases with unknown histology and the low incidence of CCA in western countries. Our group's previous study [20] estimated the proportion of HCC and CCA in patients from those with unknown histology using a multiple imputation technique and observed increasing trends of CCA in both sexes and a downward trend of HCC in males but not in females. With the existing momenta in the population, the aims of this study are to predict the gender-specific incidence trends for HCC and CCA and provide projections to the year 2030 in Songkhla, southern Thailand.

## 2. Methods

### 2.1. Registry

This study used LBD cancer cases registered at the Songkhla population-based cancer registry between 1989 and 2013. The Songkhla cancer registry covers 16 districts containing around 1.4 million people. The study protocol was approved by the Institute Ethics Committee of the Faculty of Medicine, Prince of Songkla University.

### 2.2. Cancer Cases and Population Data

The process of grouping the LBD cancers and multiple imputation technique in our previous article [20] is repeated here briefly. LBD cancer cases were grouped into four categories based on the International Classification of Diseases for Oncology, third edition (ICD-O-3) as follows: (1) hepatocellular carcinoma; (2) cholangiocarcinoma; (3) other specified carcinomas of liver; and (4) LBD cancer with unknown histology. The last group also included a random sample of cancer cases with unknown primary in the abdomen and unknown primary site, which were stratified by age group, sex, and year of diagnosis. We included these unknown primary cancer cases in group (4) because we believed that some of them were misclassified due to incomplete investigations (for example, the patient died or refused to undergo any invasive or surgical procedure). Cases with unknown histology were imputed with one of the other known histological categories according to the probability distribution of the groups among those who had known histology. The optimal percent of cases randomly selected from the unknown primary site in the abdomen to include in group (4) differed each year with an average of 12% for the period 1989 to 1997 and gradually reduced down to 0% from 2007 onwards. By 2007, high-resolution imaging allowed for very accurate diagnoses of liver cancer so that very few of these unclassified diagnoses occurred. The reason to do the complex method of multible imputation was to imitate the current situation as if the high resolution radiographic imaging had been implementing since the beginning of the registration. The sources of population denominators are described in our previous publication [20].

### 2.3. Statistical Analysis

The technique and results of multiple imputation (MI) used in this study were described in detail in our previous publication [20]. Changes to diagnostic equipment and, importantly, the change in disease classification over the time period of study affected the way doctors do radiographic and histologic investigation. In the dataset, the percentage of missing histologic confirmation gradually increased from 40% to 80% from 1989 through 2013. In that paper, the multivariate imputation by chained equation (MICE) package in R was used under the multinomial logistic regression model to fill in the histological type to the missing cases in the dataset. The variables included in the model consisted of sex, age, year of diagnosis, region, and residence district. Even though the fact of missing at random (MAR) could not be proven, we discussed that we did the prediction at the boundary of the prediction ability of the method at the 60%-67%. Even though a bias of the prediction might have occurred, it might not be avoidable since the new standard diagnostic method prevents us from getting routine tissue diagnosis.

With the final dataset in which the cases with missing histological type information were filled up by imputed data, the age-standardized incidence rates per 100,000 person-years of HCC and CCA from 1989 to 2013 were estimated by the direct method using the Segi's world standard population in each 5-year age group (age 20 to 85+ years). The trend analysis and number of LBD cancer cases by histology and sex was done by using three regression modeling strategies: joinpoint regression analysis [21], age-period-cohort (APC) modeling [22], and a modified APC modeling technique known as Nordpred modeling [23]. Joinpoint models evaluated trends from identification of statistically significant trend change points (joinpoints) and the rate of change (annual percent change) in each trend segment using a Monte Carlo permutation method. Age-period-cohort models were run in two ways to address the nonidentifiability problem. The best fitting APC model, AP, AC, or APC, for the subgroups was determined by the stepwise determination of the Akaike information criterion (AIC) which is given by −2×LL + 2×*n*, where LL is the logarithm of the likelihood estimated by the model and *n* is the number of model parameters. The models, known as AP-C and AC-P, fit the third effect (either cohort or period) to the residuals of their respective two-effect model to assess the effect of time components on the incidence trends. Nordpred models use a log-linear model with a power 5 function and 5-year periods to assess trends.

Projections were extrapolated based on the models specified above. The natural spline method was used to extend the linear and nonlinear curves of the APC models separately and then combined to obtain the projected trend. Natural splines were also used to extend the period and cohort effects into the future via nonlinear interpolation for yearly projections. For Nordpred projections, the power 5 function was used to avoid overestimation of cases from the multiplicative model through geometric attenuation of the linear drift by 21.6%, 48.3%, 65.9%, and 77.6% [24] for the first (2014–2018), second (2019–2023), third (2024–2028), and fourth (2029-2030) periods, respectively.

## 3. Results

From 1989 to 2013, there were 2,676 LBD cancer cases in the Songkhla cancer registry. The incidence in males was threefold higher than in females.  Table 1 shows the raw data for the four cancer categories previously mentioned. After the multiple imputation procedure, HCC had a larger proportion of cases than CCA. Among males, HCC accounted for a larger proportion than CCA, but among females, CCA had a higher proportion of cases than HCC.

Among males, the highest proportion of HCC cases was found in males aged 50-59 years and 60-69 years as shown in Table 2. Thus, the majority of CCA cases were males aged 50-69 years. Females had the highest proportion of HCC in the age group of 60-69 years while the highest proportion of CCA was found in those aged 70-79 years. The number of HCC cases increased with each 5-year period for both sexes whereas the CCA incidence increased as age increased. The number of cases of CCA in both sexes showed a rapid increase from 1999 to 2013.

Among males, there were two separate trends of HCC incidence from the joinpoint model. From 1989-2007, the incidence increased significantly with an annual percent change of 3.2% (95% CI: 1.3%, 5.1%) per year, after which it showed a slight decline at a rate of -2.8% per year without statistical significance (95% CI: -9.8, 4.8). The incidence of CCA in males showed a significant increase of 5.2% (95% CI: 3.8%, 6.6%) per year and, among females, the incidences of HCC and CCA gradually increased at a rate of 1.4% (95% CI: 0.0, 2.7) and 4.4% (95% CI: 3.1%, 5.7%), respectively, per year.

Table 3 shows the Akaike information criterion (AIC) values from the three age-period-cohort models for the two genders and two major LBD histologic subtypes. Among cholangiocarcinomas, the age-period-cohort (APC) model was the best model for both genders, while the age-period (AP) model was the best among males with HCC and the age-cohort (AC) model was the best among females with HCC.

**Figure 1:**
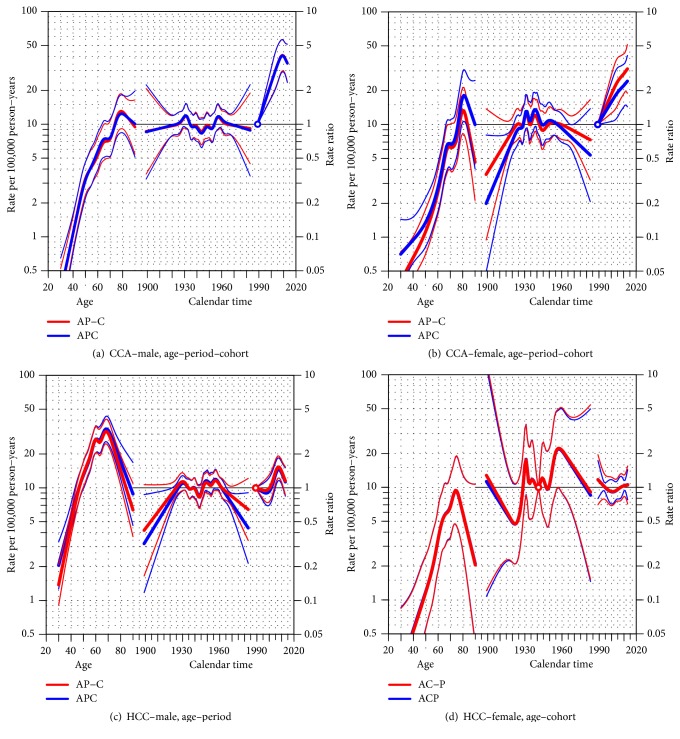
Age-period-cohort (APC) trend analysis for hepatocellular (HCC) and cholangiocarcinoma (CCA) cancers by gender; (a) CCA-male, (b) CCA-female, (c) HCC-male, and (d) HCC-female. For each group, the models with the lowest AIC value presented in Table 3 are drawn over the ones with higher AIC value. The thin lines above and below the three effects represent their 95% confidence interval.

Figure 1 shows the results of the age-period-cohort models for estimating the age-standardized incidence rates of LBD cancers during 1989–2013. Among males with CCA (Figure 1(a)), large age and period effects were seen with rates increasing for increasing age and period, although there was a slight decrease after year 2010. The cohort effect was not significant. Trends were similar for females (Figure 1(b)). Among males with HCC (Figure 1(c)), the rate increased rapidly in 2000 and sharply decreased in 2010. Among females with HCC (Figure 1(d)) a declining trend was evident in the cohorts born after 1960. Such a decline was also observed among males with HCC.

**Figure 2:**
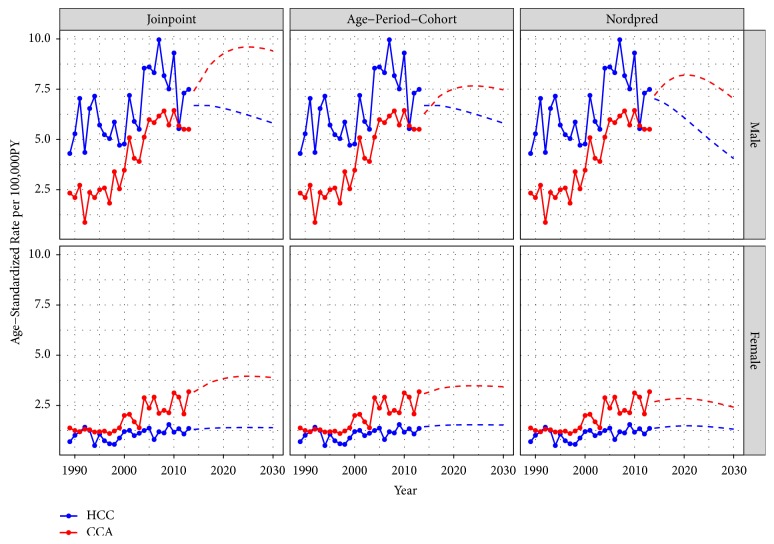
Age-standardized incidence rates of liver and bile duct cancers in Songkhla by the major histologic subtypes; hepatocellular carcinoma (HCC) and cholangiocarcinoma (CCA), using three projection models; joinpoint regression, age-period-cohort, and Nordpred, by gender in 1989-2013 and projection until 2030. PY: person-years.

**Figure 3:**
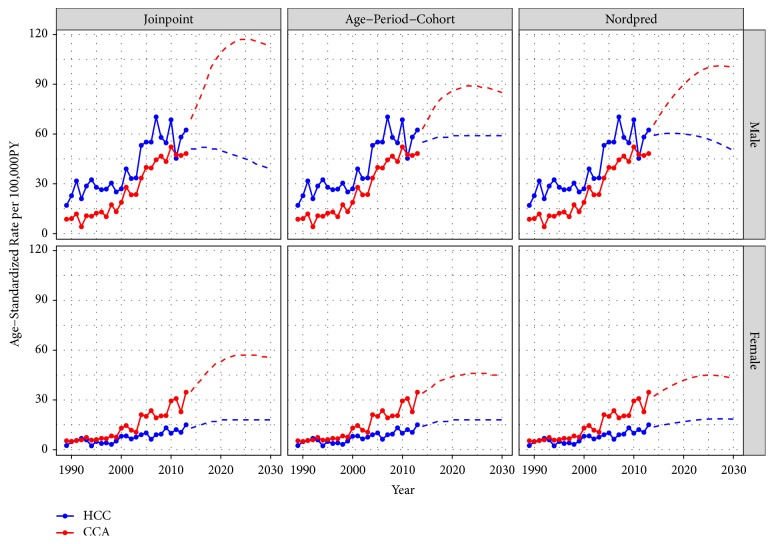
Estimated number of liver and bile duct cancers in Songkhla by the major histologic subtypes; hepatocellular carcinoma (HCC) and cholangiocarcinoma (CCA), using three projection models; joinpoint analysis, age-period-cohort, and Nordpred by sex in 1989-2013 and projection until 2030.

*Projected Trends of HCC and CCA*. Figures 2 and 3 show the trends in incidence rate and number of cases of LBD cancers in Songkhla, respectively, by histologic subtype and sex. The incidence rates of HCC among males started decreasing just before 2010 and the decrease is expected to continue in the future with attenuation of the rates. Joinpoint, APC, and Nordpred models all predicted that the rates would reach 5.8, 5.8, and 4.0 cases per 100,000 person-years, respectively (Figure 2), which can be translated to around 40-60 cases per year by 2030 (Figure 3). The incidence of male CCA is expected to increase in the future. All models forecasted that the rates would reach 9.4, 7.5, and 7.0 cases per 100,000 person-years, respectively, translating to approximately 100 cases by 2030.

In females, the incidence of HCC is expected to remain stable. By 2030, all models predict the incidence rates to reach 1.4, 1.5, and 1.3 cases per 100,000 person-years, which corresponds to about 18 cases per year. The incidence of CCA is expected to increase more rapidly than that of HCC. By 2030, the models predict the rates to reach 3.9, 3.4, and 2.4 cases per 100,000 person-years, translating to 43-55 cases annually.

## 4. Discussion

Our findings show that the incidence of HCC has been decreasing and among males will continue to decrease in the future. In females, the incidence of HCC is expected to continue to remain stable. In contrast, the incidence of CCA has been increasing in both males and females and this is expected to continue with a slight attenuation in the future. The magnitudes of the changes expected in the future vary based on the projection model. In reality, the magnitude of changes will likely be affected by future healthcare planning and other cancer control programs in southern Thailand.

Other Asian countries such as China, Taiwan, and Singapore have provided evidence of effective reductions in the incidence of HCC after implementation of universal HBV immunization [25]. Our study demonstrated that the incidence of HCC in males in southern Thailand decreased during 2007-2013 while the incidence in females remained stable during the same time period. There are a few survey reports showing that the prevalence of HBV positivity among blood donors has declined from 7.1% in 1988, to 2.6% in 2009 [26]. It has been shown that new blood donors are good proxies of the general Thai population in monitoring the seroprevalence of HBV in Thailand [27]. From that study, the pooled prevalence estimate among the general Thai population was 5.1% (95% CI: 4.3–6.0%) in 2015. With extensive use of healthy regular blood donors, the introduction of HBV vaccine in the Thai population, the by-product of the attempt to reduce HIV infection in Thailand, and other measures to reduce blood-borne viral transmission might explain this reduction. So the decrease in the incidence of HCC in Thailand, which is mainly HBV-related, will probably continue.

The HBV immunization programme in newborns in Thailand was implemented in the early 1990s, and a gradual decline in the incidence of HCC is anticipated as these cohorts enter the age of 25-30 years. However, the voluntary HBV immunization had long been adopted in the Thai population since the vaccine was first launched in the market. An evaluation of the HBV vaccination program in Bangkok showed a high prevalence of HBV immunity after 20-year vaccination in Thai children [8]. The HCC incidence significantly declined among Thai children who received hepatitis B vaccine at birth [28]. The high proportion of people who had voluntary immunization of HBV before 1992 could explain the sharp decrease in the incidence rates prior to 2010 when newborns immunized by the national HBV vaccination program reached their 18^th^ birthday. This may also explain the declining trend in the cohorts born before 1960 seen in both sexes which is much earlier than the time of the nation-wide HBV immunization among newborns.

Although aflatoxin B_1_ exposure is still prevalent in Thailand [8, 9] and there is evidence that aflatoxin is probably among the causes of HCC in Thai population [10], there has been no strategic policy to reduce aflatoxin exposure at the population level. The fact that traffic accidents have been the leading cause of deaths in young adults, especially during the long holiday vacations, may shed light on this issue. Attempts to reduce alcohol consumption have been promoted but its effects on reducing the incidence of HCC have not been demonstrated.

The trends in incidence of CCA in Thailand are largely influenced by a liver fluke,* Opisthorchis viverrini,* infestation [11]. As found in this study, CCA in Songkhla has been increasing. However, Songkhla is free of* Opisthorchis viverrini* [29]; the province is not the habitat of the* Bithynia siamensis* snail, the first intermediate host of* Opisthorchis viverrini* trematode [30].

As shown in Figures 2 and 3, a significant increase in the incidence of both HCC and CCA around 2003 and 2004 was seen in both sexes. The use of high-resolution imaging technology leading to the adoption of the Bismuth-Corlette classification of CCA around 2000 could explain the rapid rise in the incidence of both diseases. While the number of suspected cases of both diseases increased rapidly, the percentage of histologically verified cases dropped accordingly as shown in our previous study [20]. For this reason, we included a proportion of the intra-abdominal and unknown primary cancers, which in the past might not have been diagnosed as LBD cancer by this current diagnostic method, into the multiple imputation process in order to simulate the condition of higher sensitivity of the diagnostic tool than it was in the past to minimize the overestimation of the trends in recent years.

All three models used in this study demonstrate an increase in the incidence of CCA until 2030, whereas the incidence of HCC is expected to either be stable or decreasing in the future. The trends in HCC incidence correspond with the hepatitis B immunization activities in the country, while the increasing trends in CCA incidence cannot be explained by* Opisthorchis viverrini* infestation in Songkhla province. A reversal of the HCC/CCA ratio is of national and international interest as it has not been observed in areas which are not endemic with* Opisthorchis viverrini *infestations. Such a low baseline incidence of female HCC prevented the models from capturing any statistical trend in the future.

In Table 3, HCC trends in males were influenced by a cohort effect while the period effect played a smaller role. Among females, the incidence rates are explained better by the period effect and the full APC model explains the results the worst. The trends in males are consistent with reports from Taiwan [5] where national HBV vaccination in newborns was launched in 1984, eight years ahead of Songkhla. The small sample size in the youngest and oldest cohorts and periods before year 2004 may have had an effect on the level of significance. However, the conflicting significance of the cohort and period effects among males and females is minimal, as shown in Table 3 where the difference in the AIC values was modest. The adoption of the HBV vaccine in the population was some years before the launch of the national newborn vaccination program and such a voluntary immunization program is better explained by the period effect than the cohort effect. In CCA, the full age-period-cohort model gave the lowest AIC in both genders (Table 3). However, Figures 1(a) and 1(b) showed the incidence rates to be increasing in later years while decreasing in younger generations.

Thailand has been making important public health efforts to provide for its population. Along with the newborn vaccination program in 1992 [8], Thailand began universal health coverage in 2002 [31]. As CCA in southern Thailand is not related to* Opisthorchis viverrini *infestation, an effort to find controllable risk factors is crucial for this region. Future studies are needed to confirm that the baseline incidence of CCA is increasing in other regions of Thailand. The country launched the Alcohol Control Act in 2008 [32] and the enforcement has been pushed hard against traffic accident during the long holidays but the accidents never decline and the alcohol consumption control is hardly reached [33]. Such evidence does not explain the stable and the decline in HCC incidence in females and males in the future.

## 5. Conclusion

Our study suggests that, in Songkhla province, the incidence rates of LBD cancer have been increasing from 1989 and will continue to increase in the next 15 years. Cholangiocarcinomas will contribute more to this increase and will have a greater impact on the incidence rates and number of LBD cancers than hepatocellular carcinomas for both genders. The initiatives of comprehensive cancer prevention and control may play important roles in the reduction of the hepatocellular carcinoma incidence. While it is known that Songkhla is free from* Opisthorchis viverrini* infestations, the effects of migration of people from endemic areas into southern Thailand and the travelling patterns of local southern people to endemic regions may influence the prevalence of liver fluke infestation, even though the life cycle of this parasite cannot be completed locally. Other associated factors for cholangiocarcinoma in this area should also be the focus of further investigations.

## Figures and Tables

**Table 1:** Liver and bile duct cancer cases by histological type and sex before and after imputation.

Histologic subtype	All (n=2,676)				Males (n=1,947)				Females (n=729)			
	Before	%	After	%	Before	%	After	%	Before	%	After	%
HCC	409	15.4	1,188	44.4	329	16.9	1004	51.6	80	11.0	184	25.2
CCA	358	13.4	1,020	38.1	206	10.6	657	33.7	152	20.8	363	49.8
Other^†^	165	6..1	468	17.5	89	4.5	286	14.7	76	10.4	182	25.0
Unknown^‡^	1,455	54.3	-	-	1,152	59.1	-	-	303	41.6	-	-
Unknown^¶^	289	10.8	-	-	171	8.8	-	-	118	16.2	-	-

HCC: hepatocellular carcinoma, CCA: cholangiocarcinoma, ^†^liver and bile duct cancer with unspecified histology, ^‡^liver cancer with unknown histology, and ^¶^malignant neoplasms of the abdomen (C76.2) and unknown primary site (C80.9).

**Table 2:** Number and percentage of liver and bile duct cancers by gender and histologic subtype (n=2,676).

Variable	Males				Females			
	HCC (n=1,004)		CCA (n=657)		HCC (n=184)		CCA (n=363)	
	Number	%	Number	%	Number	%	Number	%
Age group (years)								
<40	91	9.1	43	6.5	17	9.2	34	9.4
40-49	176	17.5	105	16.0	25	13.6	35	9.6
50-59	298	29.7	158	24.0	39	21.2	51	14.0
60-69	280	27.9	156	23.7	53	28.8	87	24.0
70-79	141	14.0	125	19.0	41	22.3	91	25.1
≥80	18	1.8	70	10.7	9	4.9	65	17.9
Period of diagnosis								
1989-1993	122	12.2	45	6.8	25	13.6	30	8.3
1994-1998	143	14.2	64	9.7	18	9.8	33	9.1
1999-2003	158	15.7	106	16.1	36	19.6	58	16.0
2004-2008	292	29.1	204	31.1	44	23.9	104	28.7
2009-2013	289	28.8	238	36.2	61	33.2	138	38.0

HCC: hepatocellular carcinoma. CCA: cholangiocarcinoma.

**Table 3:** Akaike information criterion values for the age-period, age-cohort, and age-period-cohort models against the age-drift model.^‡^

Model	Hepatocellular carcinoma		Cholangiocarcinoma	
	Male	Female	Male	Female
Age-period	1520.5^†^	1522.4	1527.6	1519.5
Age-cohort	1520.6	1522.3^†^	1527.7	1519.5
Age-period-cohort	1521.0	1524.4	1523.8^†^	1517.3^†^

^†^The best fitting model based on the Akaike information criterion.

^‡^Relative values that weight the goodness-of-fit of the model to empirical data.

## Data Availability

The data used to support the findings of this study are available from the corresponding author upon request.
